# Multi-systemic risk and protective factors for contact with criminal justice services for vulnerable children

**DOI:** 10.23889/ijpds.v8i2.2211

**Published:** 2023-09-14

**Authors:** Anna Leyland

**Affiliations:** 1 University of Sheffield, Sheffield, United Kingdom

Children who experience adversity in childhood are more likely than their peers to have contact with criminal justice services. There are many risk and protective factors that can influence outcomes for vulnerable children, including factors within the child, family, school, community and the wider socio-political context. Little is currently understood about how these multi-systemic factors impact on individual outcomes, and how the formal response from child protection services may improve outcomes for vulnerable children. Through the use of a whole population administrative dataset and multi-level models the present findings explore the role of multi-systemic factors on later involvement with criminal justice services in England. In particular, the complex interactions between childhood adversity, ethnicity, poverty, additional educational needs and disabilities, engagement in education, and the level of school based and community child protection interventions. The research also reports on how individual outcomes are influenced by community level inequalities and public sector service resources, that differ between regions of England. The findings uphold the importance of recognition of vulnerable children as a heterogenous group whose outcomes are strongly influenced by multi-systemic factors, which could be influenced directly by policy and practice changes. The presentation also highlights the potential contribution and limits of administrative data to the field.

